# Coordinated dynamics of excitatory and inhibitory synapse assembly

**DOI:** 10.1101/2025.06.02.657384

**Published:** 2025-06-03

**Authors:** Krassimira Garbett, James Allen, Richard C. Sando

**Affiliations:** 1 Department of Pharmacology, Vanderbilt Brain Institute, Vanderbilt University, Nashville, TN 37240 USA

## Abstract

Neural circuits composed of multitudes of diverse synaptic connections self-organize during mammalian brain development. A balance between excitatory and inhibitory synaptic function is required for information processing by these neural circuits. Despite the importance of this balance, the interplay between excitatory and inhibitory synaptic assembly during circuit establishment remains unclear due to a lack of means to monitor both processes simultaneously. Here, we develop imaging approaches to visualize and track excitatory and inhibitory synapses concurrently. By applying these approaches, we find that despite continual dynamics, excitatory and inhibitory synaptic density remain at synchronized levels during synaptogenesis. These results support coordinated excitatory and inhibitory synapse assembly to maintain functional balance despite continual synaptic turnover.

## Introduction

Synapse assembly, maturation, and elimination require spatial and temporal coordination of several cell biological processes, beginning with pre- and postsynaptic recognition of combinatorial networks of cell surface receptors, adhesion molecules, and secreted factors^1^. These extracellular networks subsequently initiate bi-directional signaling cascades that stabilize nascent synapses and recruit the corresponding functional machinery essential for diverse physiological synaptic parameters. Synapse elimination likely involves termination of these signals and disassembly of trans-synaptic adhesion complexes^1,2^. While increasing evidence supports this model, the dynamics of mammalian synapse assembly, together with the interplay between excitatory and inhibitory synapses during this process, remain incompletely understood due to the paucity of approaches to visualize these events in real time^3^.

A dynamic relationship between excitatory and inhibitory synaptic function is critical for computation by neural networks. Developmental imbalances between excitatory and inhibitory synapses contribute to neurological disorders, including autism^4^. Moreover, the pathophysiology of Rett syndrome, a severe neurodevelopmental disorder, involves a reduction in spontaneous activity caused by an increase in synaptic inhibition over excitation^5^. Interestingly, a structural coordination between excitatory and inhibitory synapses and functional interactions between these synapses can maintain a constant excitatory/inhibitory ratio along the same dendritic tree^6^. This raises the hypothesis that excitatory and inhibitory synapse assembly is coordinated to maintain a functional balance during development.

Despite these advances, several obstacles towards testing this hypothesis remain. First, approaches to simultaneously visualize interactions between mammalian pre- and postsynaptic compartments in real time over relatively long timescales with high spatiotemporal resolution remain lacking. Moreover, long-term time-lapse imaging parameters for both excitatory and inhibitory synapses concomitantly are limited^7–9^. Invertebrate systems enable global and long-term time-lapse imaging of circuit assembly *in vivo*^10,11^. For example, recent advances using intravital imaging have enabled high content, long-term visualization of growth cones and filipodial dynamics during *Drosophila* visual system development^12,13^. In mammalian systems, dendritic spines, structures associated with excitatory postsynaptic compartments, can also be visualized long-term during their formation and dynamics^14^. Studies using time-lapse two-photon microendoscopy in the CA1 region of live mice provided evidence for a remarkably dynamic turnover of dendritic spines^15^. This was in contrast to studies in the neocortex, which found a higher level of spine stability, suggesting that different cell types may display different synaptic turnover rates to support their network functions^16–18^.

To help address the dynamic interplay between excitatory and inhibitory synapse assembly in mammalian neurons, we generated long-term time-lapse imaging approaches to simultaneously monitor the dynamics of presynaptic Synaptobrevin-2/VAMP2 (Syb2), excitatory postsynaptic Homer1c, and inhibitory postsynaptic Gephyrin, which are routinely used to label pre- or postsynaptic compartments. These strategies enable examination of the dynamics of excitatory and inhibitory synaptic compartments, and their co-clustering into contacts. By applying these approaches, we observed that subsets of synapses are dynamic even in mature hippocampal neurons. Despite this, excitatory and inhibitory synapse levels remain balanced during synaptogenesis. Collectively, these approaches provide new insights into the dynamics and coordination of inhibitory and excitatory synaptic connections during mammalian circuit establishment.

## Results

**Figure 1:**
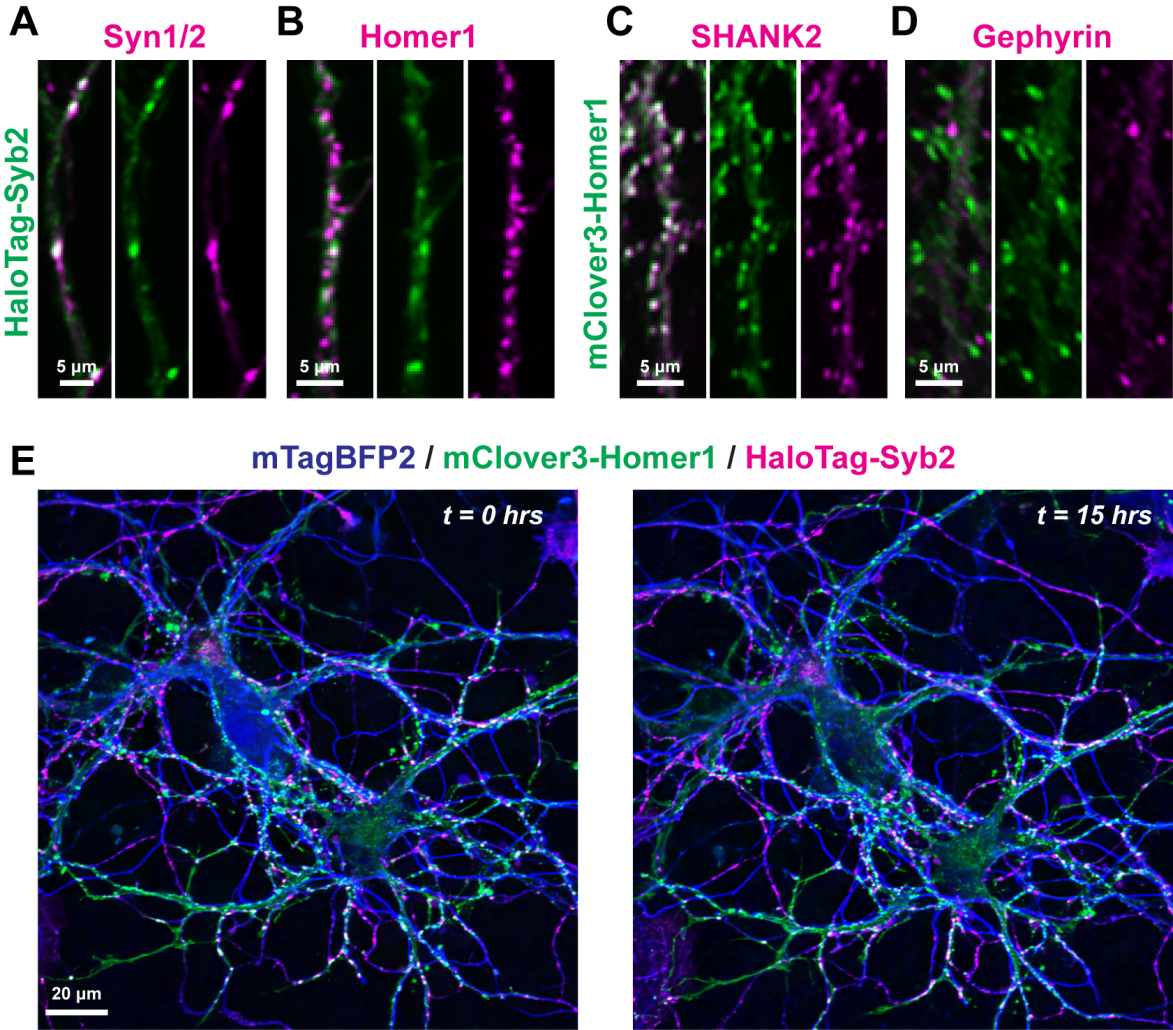
Characterization of live imaging reporters to simultaneously monitor presynaptic and excitatory postsynaptic compartments **A & B,** lentivirally delivered HaloTag-Syb2 in primary hippocampal neurons labeled with cell-permeant JF646 dye and subsequently immunostained for Syn1/2 (**A**; presynaptic) or Homer1 (**B**; excitatory postsynaptic). **C & D,** primary hippocampal neurons transduced with lentivirus encoding mClover3-Homer1c immunostained for SHANK2 (**C**; excitatory postsynaptic) or Gephyrin (**D**; inhibitory postsynaptic). **E,** representative live hippocampal neurons expressing mClover3-Homer1c, HaloTag-Syb2 and mTagBFP2 as a cell fill before (*left*) and after (*right*) a 15-hr imaging period. See Figure S1 for additional characterization of lentiviral reporters.

While canonical synaptic proteins are routinely used as markers of mature pre- or excitatory postsynaptic compartments, long-term live imaging both simultaneously has been challenging. Thus, we developed live imaging procedures to visualize excitatory synaptic dynamics over relatively long timescales (Fig. 1, S1). We started by generating a set of lentiviral reporters based on routinely used synaptic markers. Namely, these reporters included mClover3-Homer1c and HaloTag-Syb2 fusions. mClover3 is a brighter and more photostable version of the green fluorescent protein Clover^19^, and HaloTag is a self-labeling tag with a panel of versatile and highly stable fluorescent dyes available, including far red dyes^20^. We examined these reporters in primary hippocampal cultures, a widely used reduced experimental system. We initially validated the proper localization of these reporters via co-staining hippocampal neurons for additional synaptic markers (Fig. 1A–D, S1). Hippocampal neurons transduced with HaloTag-Syb2 were live labeled with cell permeant JF646 HaloTag ligand^21^, and subsequently immunolabeled for endogenous synaptic markers. HaloTag-Syb2 co-localized with presynaptic Syn1/2, and co-clustered together with postsynaptic Homer1 (Fig. 1A & B, S1A). mClover3-Homer1c exhibited higher co-localization another excitatory postsynaptic marker, SHANK2, compared to the inhibitory postsynaptic marker Gephyrin (Fig. 1C & D, S1B). These results support that these lentiviral reporters serve as reliable proxies for their respective synaptic compartments.

We next tested their compatibility and efficacy for long term time-lapse imaging. Pre- and postsynaptic combinations (HaloTag-Syb2 with mClover3-Homer1) were transduced into primary hippocampal neurons together with lentivirally delivered mTagBFP2 as a cell marker. Cells were then labeled live for JF646 HaloTag ligand as before and imaged at DIV12–14, a point when excitatory synapse functional maturation peaks in primary hippocampal neurons^22,23^. Cells were imaged for 15-hrs total at 5-min intervals in a live cell incubation chamber with resonance scanning confocal microscopy (Fig. 1E, Movie S1–3). This permitted stable imaging of labeled synaptic compartments, including co-clustered pre- and postsynaptic compartments, without detectable photobleaching.

**Figure 2:**
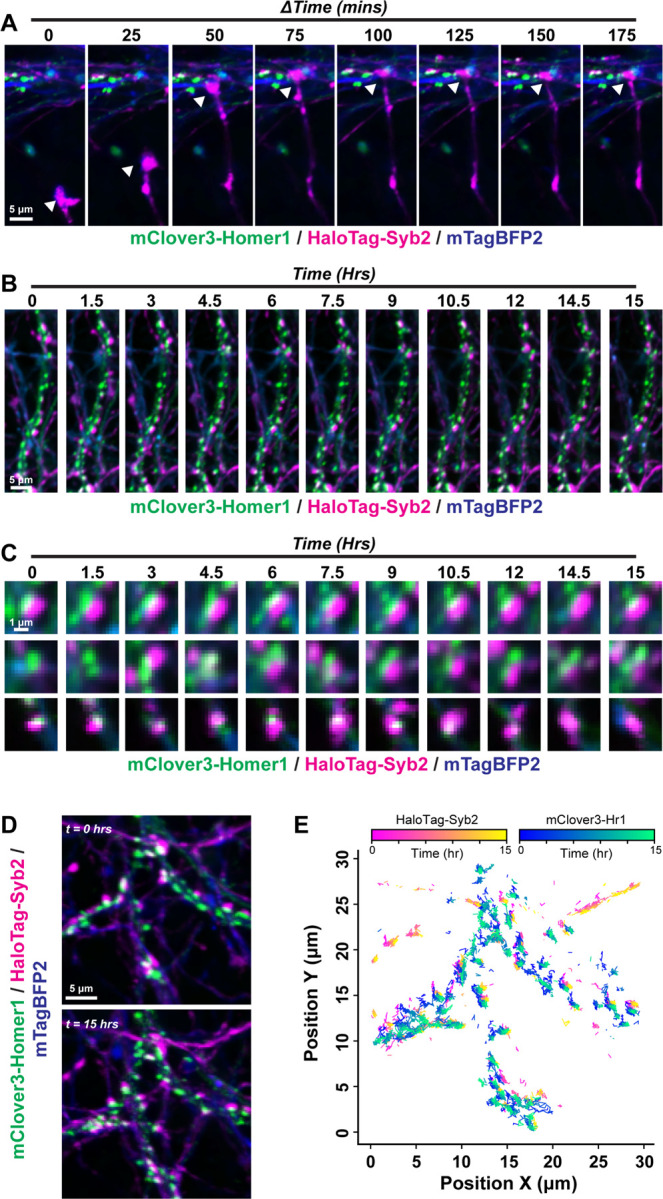
Visualization of distinct populations of excitatory synapses **A,** visualization of nascent excitatory synapses. Representative HaloTag-Syb2 labeled growth cone forming a co-cluster with a mClover3-Homer1c puncta along an mTagBFP2-filled dendrite. **B,** representative dendrite from image acquisition over a 15-hr period at 5-min intervals in DIV14 primary hippocampal neurons transduced with HaloTag-Syb2, mClover3-Homer1c and mTagBFP2. **C,** representative synaptic co-clusters that are stable, formed, or become eliminated over the 15-hr imaging session. **D & E,** tracking of excitatory synaptic puncta. **D,** representative mTagBFP2-labeled dendrites containing HaloTag-Syb2 and mClover3-Homer1 labeled excitatory synaptic puncta. **E,** particle tracking from live imaging in **D** over a 15-hr period. See Movie S1–3 for representative live imaging data.

These strategies captured nascent synapses assembling even in relatively mature primary hippocampal neurons, including HaloTag-Syb2-positive growth cones approaching and forming contacts with mClover3-Homer1c postsynaptic compartments along mTagBFP2-filled dendrites (Fig. 2A). These approaches visualized distinct populations of excitatory synapses that were stable during the 15-hr session, formed into co-clusters during imaging, or disassembled (Fig. 2B & C). We next developed a set of parameters to track and quantify individual mClover3-Homer1 puncta, HaloTag-Syb2 puncta, and Homer1:Syb2 co-clustered puncta over the 15-hr imaging period (Fig. 2D & E). Through our particle tracking, we were able to track all the pre- and postsynaptic compartments that persisted through the imaging period, as well as the compartments which existed only temporarily. These analyses highlight the dynamic nature of excitatory synapses over these timescales. Thus, these live imaging parameters enabled extended visualization and tracking of the interplay between pre- and postsynaptic compartments in live hippocampal neurons.

**Figure 3:**
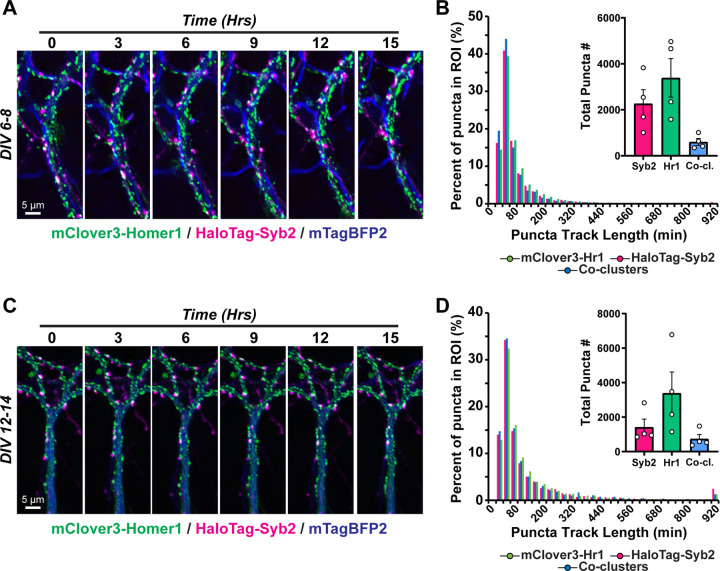
Excitatory synapse tracking in both developing and mature hippocampal neurons **A,** representative mTagBFP2-filled dendrite with HaloTag-Syb2 and mClover3-Homer1c puncta over time from DIV6–8 primary hippocampal neurons. **B,** histogram depicting the distribution of puncta track length within an ROI along an mTagBFP-filled dendrite. *Inset*, average number of puncta analyzed from 4 independent experiments. **C & D,** similar to **A & B**, except for live imaging experiments conducted at DIV12–14. Numerical data are means ± SEM or histograms from 4 independent biological replicates. See Figure S2 for additional quantification and Movie S4 and S5 for representative time-lapse imaging.

Previous studies have shown that excitatory synapses reach a peak steady state of functional maturation at approximately DIV12. Therefore, we examined excitatory synapses before (DIV6–8) and after (DIV 12–14) peak functional maturity in primary hippocampal neurons (Fig. 3, Fig. S2, Movie S4 & 5). In parallel, we developed puncta tracking quantifications to identify and follow individual puncta over time and monitor their track duration along mTagBFP2-labeled dendrites (Fig. 3, S2). Interestingly, many labeled synaptic puncta were fluid, with a majority of them exhibiting a track length between 20–120 minutes. Moreover, puncta were continuously formed or eliminated throughout the 15-hr imaging period (Fig. 3A–C, Movie S4). Similarly to DIV6–8, synapses at DIV12–14 were highly dynamic. However, there was a shift in population toward longer track duration for functionally mature neurons, suggesting increased stability of synapse assembly dynamics (Fig. S2). Therefore, these reporters and approaches enable imaging and quantification of excitatory synapses that reveal distinct properties of synapse assembly at different stages of development.

**Figure 4:**
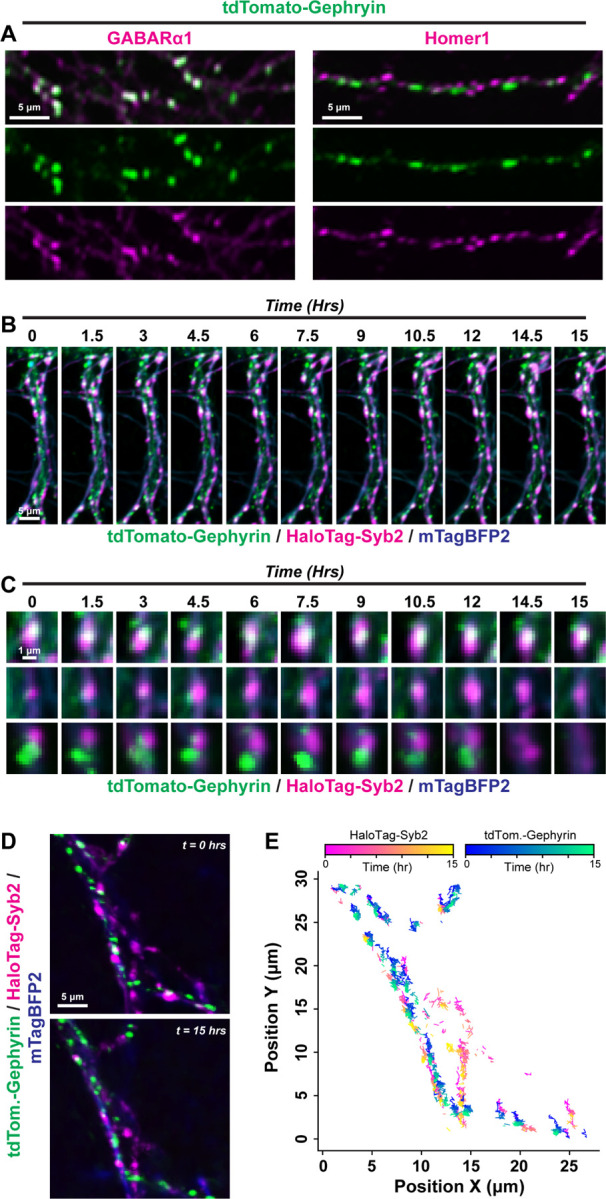
Live imaging the dynamics of inhibitory synapses **A,** co-localization of the tdTomato-Gephyrin lentiviral reporter with GABARα1 (inhibitory postsynaptic; *left*) or Homer1 (excitatory postsynaptic; *right*). **B,** representative frames from neurons expressing tdTomato-Gephyrin, HaloTag-Syb2, and mTagBFP2 as a cell marker. The same neuron was monitored over a 15-hr period with 5-min intervals between image acquisitions. **C,** representative inhibitory synaptic co-clusters that are stable, transiently formed, or become eliminated over the imaging session. **D & E,** tracking of inhibitory synaptic puncta. **D,** representative mTagBFP2-labeled dendrites containing HaloTag-Syb2 and tdTomato-Gephyrin labeled synaptic puncta. **E,** particle tracking from live imaging in **D** over a 15-hr period. See Movie S6 for representative live imaging data.

We then generated constructs to facilitate visualization and tracking of inhibitory synapses over time (Fig. 4). To enable compatibility with our other tools, we generated a tdTomato-Gephyrin fusion protein encoded in lentivirus. This fluorescent fusion protein was highly co-localized with another inhibitory postsynaptic marker, GABARα1, but not the excitatory postsynaptic marker Homer1 (Fig. 4A). We then co-infected primary neurons with tdTomato-Gephyrin, HaloTag-Syb2, and mTagBFP2, and conducted live imaging as before (Fig. 4B–E). These approaches effectively visualized presynaptic and inhibitory postsynaptic puncta, as well as synaptic co-clusters, over the 15-hr imaging period, and captured dynamic populations of inhibitory synapses, including stable, transiently formed, or eliminated (Fig. 4B & C). Similar to our analyses of excitatory synapses, we applied puncta tracking approaches, which tracked and quantified these populations over the 15-hr session (Fig. 4D & E). Altogether, this enables long-term visualization of excitatory and inhibitory synapses in primary hippocampal neurons.

**Figure 5:**
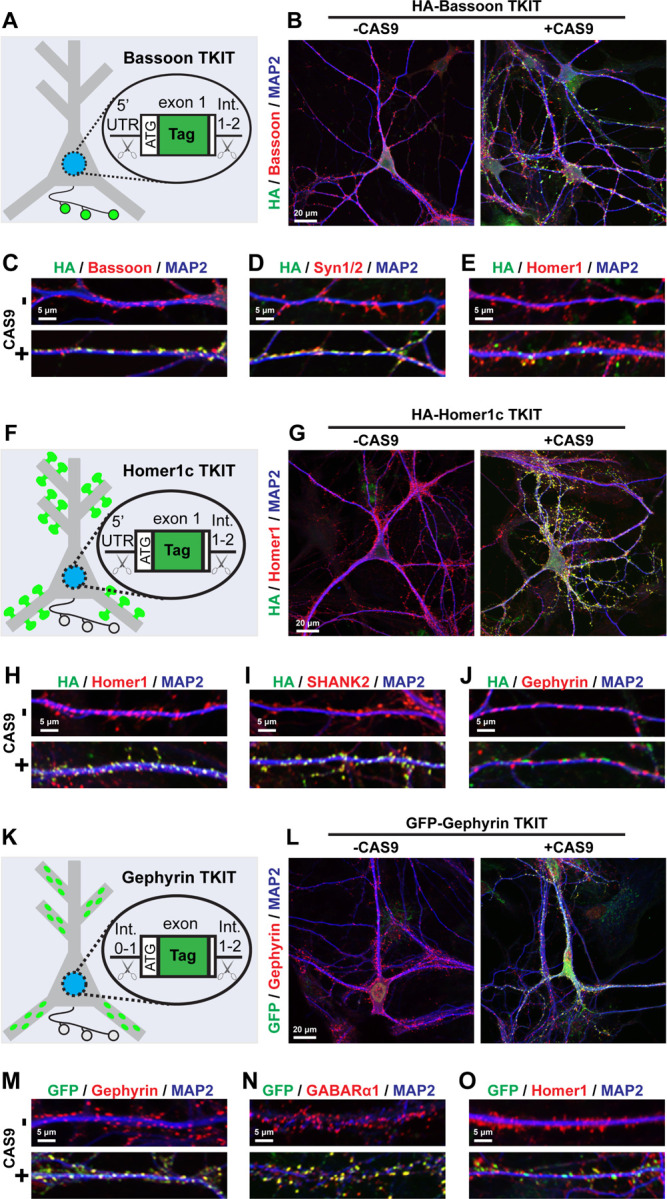
Characterization of TKIT CRISPR/Cas9-based reporters to label endogenous pre- or postsynaptic compartments **A-E,** characterization of CRISPR/Cas9 tagging strategy for endogenous presynaptic Bassoon. **A,** diagram of TKIT CRISPR/Cas9 approach to tag endogenous Bassoon via AAV-mediated delivery of TKIT CRISPR components. **B,** example neurons transduced with AAVs encoding an HA-Bassoon DNA donor, sgRNAs, without (*left*) or with (*right*) Cas9 overexpression. Neurons were co-stained for HA, endogenous Bassoon, and the somatodendritic marker MAP2. **C-E,** co-localization of HA-tagged Bassoon with endogenous Bassoon (**C**), another presynaptic marker Syn1/2 (**D**), and the excitatory postsynaptic marker Homer1 (**E**). **F-J,** TKIT tagging of endogenous postsynaptic excitatory Homer1c. **F,** diagram of the CRISPR/Cas9-mediated tagging strategy for endogenous Homer1c. **G,** example primary hippocampal neurons transduced with AAVs encoding the HA-Homer1c DNA donor and sgRNAs without (*left*) or with (*right*) co-delivery of Cas9-encoding AAVs. Neurons were co-stained for HA tag, endogenous Homer1, and MAP2. **H-J,** co-localization of HA-tagged Homer1c with endogenous Homer1 (**H**), another excitatory postsynaptic marker SHANK2 (**I**), and the inhibitory postsynaptic marker Gephyrin (**J**). **K-O,** validation of TKIT-based tagging of endogenous inhibitory postsynaptic Gephyrin. **K,** diagram of endogenous Gephyrin tagging via TKIT CRISPR/Cas9. **L,** example primary hippocampal neurons transduced with AAVs encoding the Gephyrin tagging donor and sgRNAs without (*left*) or with (*right*) co-delivery of Cas9-encoding AAVs. Neurons were co-stained for GFP, endogenous Gephyrin, and MAP2. **M-O,** immunostaining for GFP-tagged Gephyrin together with endogenous Gephyrin (**M**), another inhibitory postsynaptic component GABARα1 (**N**), and the excitatory postsynaptic marker Homer1 (**O**). See Figures S3 and S4 for additional characterization of TKIT CRISPR/Cas9 tagging approaches.

Given these reporters rely on overexpression of the fluorescently-labeled proteins, as a parallel approach, we also utilized recent advances in CRISPR/Cas9 knock-in approaches to introduce tags into genomic loci within postmitotic neurons^24^ (Fig. 5 and Fig. S3 & S4). We employed the TKIT (tandem knock-in with two guides) approach that enables introduction of tags into endogenous neuronal genomic loci with high efficacy^24^. Moreover, the TKIT labeling approach avoids the concern of variable indels in coding regions following CRISPR modification as observed with other approaches^25,26^. Consistent with these studies, we found that this system robustly tagged endogenous GluA2 in primary hippocampal neurons (Fig. S3A & B). Since we are focused on developing tagging strategies for commonly used pre- and postsynaptic markers, we implemented TKIT strategies for presynaptic Bassoon, excitatory postsynaptic Homer1c, and inhibitory postsynaptic Gephyrin, and generated AAVs encoding the respective sgRNAs/donor or Cas9.

We first tagged Bassoon in primary hippocampal cultures with an N-terminal HA tag (Fig. 5A–E, Fig. S4A). When we virally transduced cultures with HA-Bassoon donor and sgRNAs, we detected robust HA labeling only when Cas9 was also co-delivered. At DIV12–14, the HA staining overlapped with the staining for endogenous Bassoon (Fig. 5B & C). Endogenously tagged HA-Bassoon also co-localized with presynaptic Syn1/2, and formed co-clusters with postsynaptic Homer1 (Fig. 5D, E, Fig. S4A). Primary neurons harboring TKIT AAVs for HA tagging of Homer1c also displayed robust HA staining that co-localized with endogenous Homer1c only when Cas9 was co-delivered (Fig. 5G & H). Furthermore, HA-Homer1c co-localized with another excitatory postsynaptic protein, SHANK2, but was excluded from inhibitory postsynaptic sites detected by staining for Gephyrin (Fig. 5I, J, Fig. S4B). We used an analogous approach to tag endogenous Gephyrin with GFP, which was based on the design of Fang *et al*., 2020 but with further optimized sgRNAs. As expected, tagged Gephyrin co-localized to inhibitory synapses with high-fidelity and was excluded from excitatory synapses (Fig. 5K–O, Fig. S4C). Namely, GFP-tagged Gephyrin co-localized with endogenous Gephyrin and GABARα1, but not excitatory Homer1 (Fig. 5M–O, Fig. S4C). Tagged mRNA transcripts were only detected when donor/sgRNA AAVs was co-delivered with Cas9 (Fig. S4D-F). Moreover, we estimated the overall efficiency of Homer1c and Gephyrin TKIT approaches using standard curve RT-qPCR, and found they modify approximately 5% of total transcripts (Fig. S4G). These CRISPR/Cas9 approaches build upon the original TKIT toolbox and provide systems to introduce tags and label endogenous presynaptic and postsynaptic proteins.

**Figure 6:**
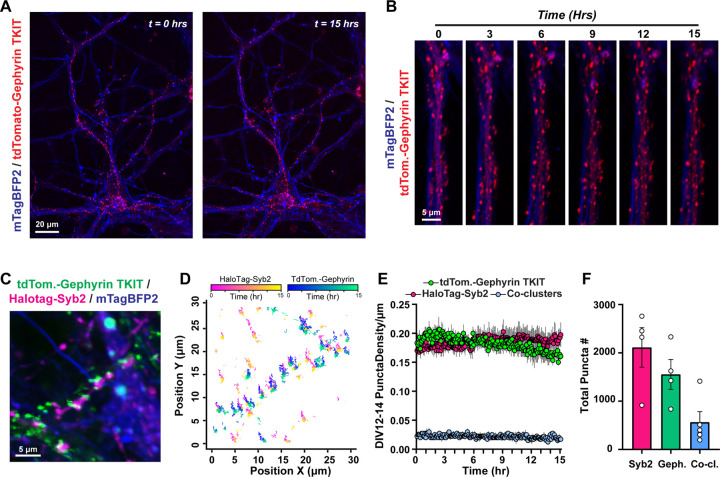
Long-term imaging of endogenous inhibitory postsynaptic Gephyrin **A,** example primary hippocampal neuron harboring tdTomato-tagged endogenous Gephyrin before and after a 15-hr imaging period. mTagBFP2 expressed via lentivirus was used as a cell marker. **B,** example dendrite with endogenously labeled tdTomato-Gephyrin time-lapse imaged over a 15-hr period at 5-min intervals. **C & D,** simultaneous visualization (**C**) and tracking (**D**) of endogenous Gephyrin together with HaloTag-Syb2 labeled presynaptic terminals. Primary hippocampal neurons were transduced with AAVs enabling TKIT tagging of Gephyrin with tdTomato together with lentiviruses encoding mTagBFP2 and HaloTag-Syb2. **E,** density of tdTomato-Gephyrin TKIT puncta, HaloTag-Syb2 puncta, or Gephyrin/Syb2 co-clusters across mTagBFP2-filled dendrites over time. **F,** average number of puncta analyzed from 4 independent experiments. Numerical data are means ± SEM from 4 independent biological replicates. See Figure S5 for additional quantification and Movie S7 and S8 for representative time-lapse imaging.

We subsequently used a new Gephyrin donor DNA to label this inhibitory postsynaptic protein with tdTomato for live imaging (Fig. 6, Fig. S5). We then conducted time-lapse imaging as before in CRISPR-modified neurons co-transduced with mTagBFP2 cell fill at DIV12–14. Endogenously labeled Gephyrin puncta were monitored continuously for 15-hrs at 5-min intervals without appreciable photobleaching (Fig. 6A & B). Consistent with lentiviral reporters, these markers allowed us to detect distinct subpopulations of synapses, some of which were stable over the imaging session while others were highly dynamic (Fig. 6B). We subsequently tested the compatibility of lentiviral reporters with TKIT reporters. We introduced tdTomato-Gephyrin TKIT AAVs together with lentiviral HaloTag-Syb2 to simultaneously label presynaptic terminals and endogenous postsynaptic inhibitory compartments (Fig. 6C–F, Fig. S5). This enabled visualization and tracking of Syb2:Gephyrin co-clusters together with quantification of their density and mobility along mTagBFP2-labeled dendrites over time (Fig. 6C–F, Fig. S5A & B). Collectively, this molecular toolbox highlights the dynamic nature of excitatory and inhibitory synapses in both developing and relatively established primary hippocampal neurons.

**Figure 7:**
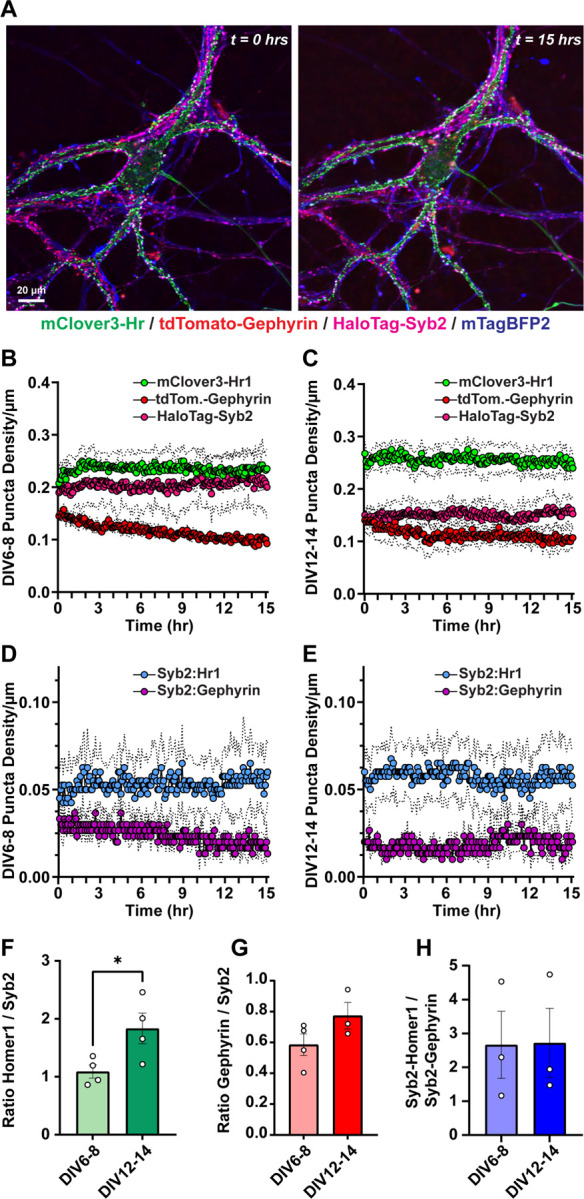
Simultaneous visualization of excitatory and inhibitory synapse dynamics during synaptogenesis **A,** representative primary hippocampal neurons expressing mClover3-Homer1, tdTomato-Gephyrin, HaloTag-Syb2, and mTagBFP2. **B,** average density of indicated synaptic puncta over a 15-hr imaging period from DIV6–8 primary hippocampal neurons. **C,** similar to **B**, except for the same hippocampal cultures analyzed at DIV12–14. **D,** comparison of Syb2:Homer1c and Syb2:Gephyrin co-cluster density over 15-hr imaging periods at DIV6–8. **E,** similar to **D**, except for the same hippocampal cultures analyzed at DIV12–14. **F,** quantification of the ratio of mClover3-Homer to HaloTag-Syb2 puncta at different time points. **G,** similar to **F**, except for the ratio of tdTomato-Gephyrin to HaloTag-Syb2 puncta in the same cultures. **H,** quantification of the ratio of excitatory co-clusters to inhibitory co-clusters at different time points. Numerical data are means ± SEM from 4 independent biological replicates. Statistical significance was assessed with a two-tailed t-test (*, p<0.05).

We next analyzed excitatory and inhibitory synapses concurrently in neurons transduced with mClover3-Homer1, tdTomato-Gephyrin, HaloTag-Syb2, and mTagBFP2 lentiviral reporters (Fig. 7A). We infected neurons at DIV1, and imaged the same cultures at DIV6–8 and DIV12–14 as before, and quantified the density of individual synaptic puncta as well as Syb2:Homer1c excitatory co-clusters and Syb2:Gephyrin inhibitory co-clusters over time. The presynaptic compartments labeled by HaloTag-Syb2 became less abundant as neurons matured, suggesting an elimination of presynaptic terminals (Fig. 7B & C). Despite this reduction in presynaptic inputs, the density of excitatory and inhibitory synaptic co-clusters remained at a steady state (Fig. 7D & E). Moreover, the density of inhibitory puncta we observed with these approaches aligned with our Gephyrin TKIT analyses (Fig. 6). Given these observations, we next quantified the ratio of total Homer1c:Syb2 puncta or Gephyrin:Syb2 puncta, together with the ratio of excitatory to inhibitory co-clustered puncta at different times (Fig. 7F–H). While the ratio of postsynaptic to presynaptic compartments increased over time, the overall ratio of excitatory to inhibitory synapses remained unchanged (Fig. 7F–H). These results suggest that despite the dynamic environment, hippocampal neurons maintain a balanced equilibrium of excitatory and inhibitory synapses over time.

## Discussion

Using live imaging approaches, we observed the interplay between pre- and postsynaptic compartments in primary neuron cultures over extended time periods. These results indicate that subsets of synapses are dynamic and continue to turnover even in relatively mature hippocampal neurons. Despite this turnover, excitatory and inhibitory synapses remain at a relative equilibrium over time. We postulate that intrinsic mechanisms synchronize excitatory and inhibitory synapse assembly during circuit development. Two potential models may explain this synchronization. Temporal coordination of intrinsic genetic programs may underlie synchronized assembly. Alternatively, there may be crosstalk between different synaptic subtypes in *trans* that maintains balance despite continual dynamics. These mechanisms may help balance excitation/inhibition and support the functional outputs of neural circuits.

Our approaches also display several inherent limitations which will require future advances to overcome. While we focused on canonical and widely used presynaptic and postsynaptic markers as proxies for synaptic compartments, our approaches do not assess the physical adhesive interaction between pre- and postsynaptic compartments. Combining our approaches with other systems relying on adhesion between pre- and postsynaptic cell adhesion molecules, such as SynView, GRASP, or SynapseShot^27–29^, will be important towards understanding further details of the dynamics of synapse assembly. Our approaches complement those based on trans-synaptic adhesion, considering the latter systems only exhibit signal after cell adhesion molecules contact, while our reporters can visualize synaptic components prior to forming a synapse. More generally, our observations support that some of the functional components of synapses are present in nascent pre- or postsynaptic compartments prior to their contact. Future studies combining our approaches with trans-synaptic adhesion reporters or genetically-encoded activity indicators including GEVIs^30,31^ will help unravel the temporal sequences of synapse assembly and maturation.

Our live imaging was conducted in neuronal culture given its broad applicability and tractability for molecular studies. While this system provides a powerful and widely used reduced model for mechanistic questions, future advances will be necessary to employ these tools in combination *in vivo*. Considering our approaches relied on reporter expression or CRISPR/Cas9 genome modifications, both of which require time for protein buildup to detectable levels, we were unable to visualize the very early stages of postnatal synaptogenesis occurring shortly after culturing at DIV0. Synapses are thought to form in excess during postnatal development, followed by refinement and elimination of connections^32^. Future advances will be required to assess these early events of postnatal synaptogenesis.

Our work establishes live imaging parameters that enable visualization of pre- and postsynaptic dynamics concurrently at high spatiotemporal resolution. Using these approaches, we observed that excitatory and inhibitory synapses are dynamic yet maintain a balanced equilibrium over time. Collectively, this work provides additional insights into the temporal aspects of how diverse synaptic connectivity generates synaptic networks.

## Methods

### RESOURCE AVAILABILITY

#### Lead contact

Further information and requests for resources and reagents should be directed to and will be fulfilled by the lead contact, Richard C. Sando (richard.sando@vanderbilt.edu).

#### Materials availability

All materials generated in this study will be openly shared upon request free of charge.

### EXPERIMENTAL MODELS AND SUBJECT DETAILS

#### Mice.

C57BL/6J (Jax #000664) mice were used in this study. Mice were housed in groups of 2 to 5 on a 12 h light/dark cycle with food and water *ad libidum* at the Vanderbilt Animal Housing Facility managed by the Division of Animal Care. All procedures conformed to National Institutes of Health Guidelines for the Care and Use of Laboratory Mice and were approved by the Vanderbilt University Administrative Panel on Laboratory Animal Care. Primary hippocampal cultures were generated from P0 pups.

#### Cell Lines.

HEK293T cells (ATCC # CRL-11268) were maintained in DMEM (Gibco Cat# 11995065) containing 10% FBS (Gibco Cat# 16000044), 1X Penicillin-Streptomycin (Corning Cat# MT30002Cl) at 37°C and 5% CO_2_ for a maximum of 25 passage numbers.

#### Primary hippocampal cultures.

For immunocytochemistry, hippocampal neurons were plated on a PDL-coated glass coverslip (#0, 12 mm, Carolina Biological Supply Company #633009) in 24-well plates (Genesee, cat # 25–107MP). PDL coating was accomplished using 50 mg/ml Poly-D-lysine (Gibco, Cat # A38904–01) coated for 1 hr to overnight at 37°C, followed by three washes with sterile dH_2_O and drying for 15 mins at RT. Mouse hippocampi were dissected from newborn mice and neurons were dissociated by papain (Worthington Biochemical Corporation, cat # LS003126) digestion for 20 min at 37° C, filtered through a 70 μm cell strainer (Corning, cat # 431751), and plated at density of 80,000 cells per dish/well. Plating media contained 5% fetal bovine serum (Life Technologies, cat # 16000044), B27 (Gibco, cat # 17504044), 1:50), 0.4% glucose, and 2 mM glutamine in 1x MEM. Culture media was exchanged 24 hr later (at DIV1) to growth medium, which contained 5% fetal bovine serum, B27, 2 mM glutamine in Neurobasal A (Gibco, cat # 10888022). Cytosine β-D-arabinofuranoside (Sigma, cat# C6645) was added to a final concentration of 2 μM on DIV3 in a 50% growth media exchange. Primary hippocampal cultures were infected with respective lentiviral/AAV conditions at DIV1, and imaging experiments were conducted at DIV6–16.

#### Plasmids.

Live imaging lentiviral reporters were encoded in a 3^rd^ generation lentiviral shuttle vector driven by the rat Synapsin promoter. The mClover3-Homer1c fusion contained mClover3 fused to the N-terminus of *Rattus norvegicus* Homer1 (NP_113895.1) separated by a glycine-serine linker (GGSGGGSGG). HaloTag-Syb2 was composed of HaloTag7 fused to the N-terminus of *Mus musculus* Syb2 (NP_033523.1) separated by a glycine-serine linker sequence (GSGGGG). The tdTomato-Gephyrin fusion contained tdTomato fused to the N-terminus of *Rattus norvegicus* Gephyrin (NP_074056.2) with a glycine-serine linker (GGSGGGSGG). TKIT plasmids were encoded in an AAV2 backbone harboring the tagged donor sequence together with a dual U6 promoter cassette delivering both sgRNAs. The donor sequence was designed as “flip and switch” as in Fang *et al*., 2021. Each sgRNA was driven by a U6 promoter and followed by a gRNA scaffold sequence. All molecular cloning was conducted with the In-Fusion Assembly system (Takara #638948).

#### CRISPR Tag Knockin Design.

Viral-based CRISPR tag knockins were designed according to the TKIT (Targeted KI with Two Guides) strategy in Fang *et al*., 2021^24^. The SEP-GluA2 TKIT vector was a kind gift from Drs. Richard Huganir and Alexei Bygrave. The SEP-GluA2 TKIT donor was subcloned into an AAV2 vector, and the same AAV2 vector was used for Homer1c, Gephyrin, and Bassoon KI constructs. sgRNAs were designed using the CRISPR sgRNA design tool from Integrated DNA Technologies. See Table 1 for all sgRNA sequences. For HA-Homer1c tagging, sgRNAs were designed to *Mus musculus* Ensembl variant 203 with a focus on the 5- UTR and intron 1–2. For the Homer1c donor fragment, the HA tags were placed immediately following the start ATG. For HA-Bassoon tagging, the HA tag was inserted onto the N-terminus of Bassoon using a donor harboring HA immediately after the start ATG. For GFP-Gephyrin or tdTomato-Gephyrin tagging, the donor fragment was designed to insert the tag on the N-terminus of Gephyrin. CRISPR tagging vectors were cloned into an AAV2 backbone containing dual U6 promoters driving sgRNA1 and sgRNA2 for each target along with the donor tagging insert in a ‘flip-and-switch’ orientation as in Fang *et al*., 2021^24^, flanked by sgRNA sites. AAV encoding untagged spCas9 driven by the nEF promoter was used for all CRISPR modification experiments.

#### Antibodies.

The following antibodies and reagents were used at the indicated concentrations: anti-HA rabbit (Cell Signaling Technologies, cat# 3724, 1:1,000), anti-HA mouse (Covance Cat# MMS101R; 1:1,000), anti-GFP rabbit (Life Technologies, cat# A11122, 1:1,000), anti-Bassoon mouse (AbCam, cat # L124–59, 1:1000), anti-Homer1 rabbit (Synaptic Systems, cat# 160 003, 1:5,000), anti-MAP2 chicken (EnCor Biotechnology, cat# CPCA-MAP2, 1:5,000), anti-SHANK2 guinea pig (Synaptic Systems, cat# 162204, 1:2,000), anti-Syn1/2 rabbit (Synaptic Systems, cat# 106 002, 1:5,000), anti-GABARα1 rabbit (Synaptic Systems, cat# 224203, 1:1,000), anti-Gephyrin mouse (Synaptic Systems cat# 147111, 1:2,000), anti-vGLUT1 (Millipore cat# AB5905, 1:1,000), corresponding fluorescently-conjugated goat secondary antibodies from Life Technologies (1:1,000).

#### Live imaging.

For live imaging, hippocampal neurons were cultured in 35 mm culture dishes (MatTek Corporation, cat # P35G1.514C) with PDL-coated 1.5 mm glass bottom as described above at a density of 70,000 cells/dish in 1 mL of Growth Media. Prior to imaging, a pulse of 0.2 μM JF646 Janelia Fluor^®^ HaloTagTag^®^ Ligand (Promega #GA1120) in 300 μL of the conditioned growth medium was applied to the cells for 2 hrs, followed by returning the cells back to the remaining 700 μL conditioned media. Images were acquired using a Nikon A1-R Eclipse Ti confocal microscope in resonant scanning mode with a 60x objective (Nikon #MRD01605, CFI60 Plan Apochromat Lambda, N.A. 1.4) and perfect focus operated by NIS-Elements AR acquisition software. Laser intensities and acquisition settings were established for individual channels using optimal LUT settings. Live images were acquired using multipoint acquisition to collect several cells/dish in a 1 μm Z-stack every 5 min at 4X averaging during a 15 hr period (181 time frames total per cell) while normal growth conditions were maintained (37°C, 5% CO_2_, 90% humidity) using a Tokai Hit stage incubator (Tokai Hit STXG Incubation System).

#### Image analysis.

Analysis was performed via NIS Elements. Prior to analyses, images from each cell were separated (Split Multipoints) and transformed to 2D (Unique Extended Depth of Focus (EDF)). In the NIS-Elements software, the synaptic compartments were identified as bright spots with ~1 μm diameter, and the clusters with overlapping pre- and postsynaptic compartments were considered co-clustered synapses. GA3 analyses were used to count the total number of synaptic compartments/synapses per time frame. The NIS-Elements tracking function was used to estimate the number of timeframes an individual synaptic compartment/synapse lasted. A bright spot was considered persisting even if it disappeared for ≤3 timeframes (15 min), before it reappeared again in the same location. In addition, a fluctuation of one standard deviation from the position in the previous time frame was allowed. The NIS-Elements tracking algorithm is based on Jaqaman, K. *et al*., *Nature Methods* 2008^33^. The algorithm generates frame-to-frame object linking, which works with a set of active tracks. An active track is established in the first frame. Then the algorithm goes through all of the time frames and extends the track. If there is no suitable link a gap is introduced. We used a Gap Closing of 3, which allows the algorithm to link until a gap length of 3-time frames, after which the track is terminated. A new puncta may initiate a new active track at any time during the imaging period as well. For analysis of Pearson’s correlation coefficient, the colocalization feature of NIS Elements was used to automatically calculate PCC for ROIs containing primary and secondary dendrites.

#### RT-qPCR.

Total RNA was isolated from cultured neurons at DIV14 using RNAqueous^™^-Micro kit (Invitrogen, AM1931), and then used for cDNA synthesis with Super Script IV (Invitrogen, cat # 18090050) with random hexamers. qPCR with PowerUp SYBR Green Master Mix (Applied Biosystems, cat # A25742) was used for quantification of tagged transcripts and total (tagged + non-tagged) transcripts, and the housekeeping gene GAPDH. The quantification was done by generating a standard curve with plasmids harboring the donor DNA to calculate copy number. The primer pairs are listed in Table 1.

#### Immunocytochemistry.

Cover glass (#0, 12 mm, Carolina Biological Supply Company #633009) was placed into 24-well plates and coated for 2 hrs with 100 μL of 50 μg/mL poly-D-lysine (Gibco #A38904–01) in the 37°C tissue culture incubator. Excess poly-D-lysine was removed, coverslips were washed 3x with sterile ddH_2_O and dried for 30 mins. Neurons were plated at a density of 70,000 cells/well. At DIV10–14, cells were washed briefly once with PBS, fixed with 4% PFA (Electron Microscopy Science Cat# 15714)/4% sucrose/PBS for 20 min at 4°C, and washed 3 x 5 mins in PBS. Samples were permeabilized in 0.2% Triton X-100/PBS for 5 mins at room temperature and then transferred to blocking buffer (4% BSA (Sigma Cat# 10735086001)/3% normal goat serum (Jackson Immunoresearch #005000121)/PBS). Samples were incubated in blocking buffer for 1 hr, and subsequently incubated with diluted primary antibody in blocking buffer for 2 hrs at room temperature. Samples were then washed 5 x 5 mins in PBS, incubated with fluorescently conjugated secondary antibodies diluted in blocking buffer for 1 hr at room temperature, washed 5 x 5 mins in PBS and mounted mounted on UltraClear microscope slides (Denville Scientific Cat# M1021) using 10 μL ProLong Gold antifade reagent (Invitrogen, #P36930) per coverslip. Samples were dried at RT in the dark prior to imaging.

#### Lentivirus production for culture experiments.

Lentiviruses were packaged in HEK293T cells from ATCC (CRL-11268). For lentiviral production, co-transfection of the expression shuttle vector and the three helper plasmids (pRSV-REV, pMDLg/pRRE and vesicular stomatitis virus G protein (VSVG)) was done with FuGENE6 (Promega E2691) using 2.5 μg of each plasmid per 9.6 cm^2^. Lentiviral-containing medium was collected 48 hr after transfection, briefly spun down 5,000 xg for 5 mins for removal of cellular debris and then stored at 4°C. The LV genomic titer was estimated using PowerUp^™^ SYBR^™^ Green Master Mix for qPCR (Applied Biosystems, A25742) with the following primers: F - ccactgctgtgccttggaatgc, and R - aatttctctgtcccactccatccag. Shuttle plasmids at 10x serial dilutions (1x10^5^ − 1x10^9^ copies/ml) were used for generating a standard curve. After quantification, the LVs were directly applied to primary neuron culture medium.

#### Adeno-associated virus production.

For production of AAVs, five 10-cm^2^ plates of HEK293T cells at 90% confluency were transfected via the calcium phosphate method. For transfections, 100 μg of each plasmid (pHelper, pDJ, and AAV shuttle plasmid) was mixed to a volume of 6.75 mL dH_2_O, and 0.75 mL of 2.5 M CaCl_2_ was added. The DNA/CaCl_2_ mixture was added dropwise to 7.5 mL 2× HBS, pH 7.05 (274 mM NaCl, 10 mM KCl, 1.5 mM Na_2_HPO_4_, 7 H_2_O [dibasic], 12 mM dextrose, and 42 mM HEPES) while vortexing gently. The DNA/CaPO4 mixture was incubated at room temperature for 20 min, and then 3 mL was added dropwise to each plate. Cells were washed 1× with prewarmed PBS 24 hr after transfection, and medium was replaced with fresh complete DMEM (DMEM + 10% FBS + 1X Pen/Strep.). Cells were harvested 72 hrs after transfection by 1× wash with PBS followed by addition of dissociation buffer (PBS/10 mM EDTA). A cell scraper was used to facilitate detachment, and cell suspensions were subsequently centrifuged at 1,500×g for 15 min at 4°C. Cell pellets were resuspended in 4 mL freezing buffer (150 mM NaCl, 20 mM Tris, pH 8.0, and 2 mM MgCl2), snap-frozen in 70% ethanol/dry ice for 15 min, and rapidly thawed at 37°C. After three subsequent rounds of freeze/thaws, the cell suspension was incubated in 50 U/mL Benzonase nuclease (Sigma; Cat# E1014) for 30 min at 37°C. Samples were subsequently centrifuged at 3,000×g for 30 min. Supernatant was applied to the surface of an iodixanol gradient (15%, 25%, 40%, and 60%) and ultracentrifuged at 80,000×g for 2 hr at 4°C in Seton Scientific Polyclear thin walled centrifuge tubes (7030). The 40% iodixanol gradient was harvested by puncturing the side of the tube with a sterile needle attached to a 10 mL syringe, added to 10 mL PBS/1 mM MgCl2, and concentrated in centricon concentrating tubes (100,000 MWCO; Millipore; UFC0910024), which were equilibrated with PBS/MgCl2. After 3 subsequent washes with 1x DMEM (Gibco Cat# 11995065), samples were concentrated to 100 μL, aliquoted, and stored at −80°C. The AAV particle number was estimated using PowerUp^™^ SYBR^™^ Green Master Mix for qPCR (Applied Biosystems, A25742) with primers targeting the ITR: F - ggaacccctagtgatggagtt, and R – cggcctcagtgagcga (AddGene). Packaged shuttle plasmid 10x serial dilutions (1x10^5^ − 1x10^9^ copies/mL) were used for generating a standard curve.

#### Confocal Imaging of fixed samples.

Images were acquired using a Nikon A1r resonant scanning Eclipse Ti2 HD25 confocal microscope with a 10x (Nikon #MRD00105, CFI60 Plan Apochromat Lambda, N.A. 0.45), 20x (Nikon #MRD00205, CFI60 Plan Apochromat Lambda, N.A. 0.75), and 60x (Nikon #MRD01605, CFI60 Plan Apochromat Lambda, N.A. 1.4) objectives, operated by NIS-Elements AR v4.5 acquisition software. Laser intensities and acquisition settings were established for individual channels and applied to entire experiments, and images were collected at the following resolution: 10x −1.73 μm/pixel, 20x − 0.62 μm/pixel, 60x − 0.23 μm/pixel. Image analysis was conducted using Nikon Elements and ImageJ. Brightness was adjusted uniformly across all pixels for a given experiment for Figure visualization purposes. Images were pseudocolored for Figure visualization purposes. Quantification of fluorescence intensities was conducted by imaging 3–5 image frames per biological replicate, which were averaged to generate a single biological replicate value. The averaged value for each replicate is depicted as open circles in each graph.

### QUANTIFICATION AND STATISTICAL ANALYSIS

#### Statistics.

All data are expressed as means ± SEM and represent the results of at least three independent biological replicates, as indicated within each Figure Legend and as open circles within bar graphs. Statistical significance was determined using the two-tailed Student’s t-test, one-way ANOVA with following *post hoc* Tukey tests for multiple comparisons, or two-way ANOVA with following *post hoc* Tukey tests for multiple comparisons, as indicated in the Figure Legends. Data analysis and statistics were performed with Microsoft Excel, GraphPad Prism 8.0 and GraphPad Prism 9.0.

## Figures and Tables

**Table:** RESOURCE TABLE

REAGENT or RESOURCE	SOURCE	IDENTIFIER
**Antibodies and imaging reagents**		
Anti-HA rabbit	Cell Signaling Technologies	3724
Anti-GFP rabbit	Life Technologies	A11122
Anti-Bassoon mouse	AbCam	L124-59
Anti-Homer1 rabbit	Synaptic Systems	160003
Anti-MAP2 chicken	Encor	CPCA-MAP2
Anti-SHANK2 guinea pig	Synaptic Systems	162204
Anti-Syn1/2 rabbit	Synaptic Systems	106002
Anti-Gephyrin mouse	Synaptic Systems	147111
Anti- GABARα1 rabbit	Synaptic Systems	224203
Anti-vGLUT1 guinea pig	Millipore	AB5905
Goat anti-mouse Alexa Fluor 488	ThermoFisher	A11001
Goat anti-mouse Alexa Fluor 546	ThermoFisher	A11003
Goat anti-mouse Alexa Fluor 647	ThermoFisher	A21236
Goat anti-rabbit Alexa Fluor 488	ThermoFisher	A11034
Goat anti-rabbit Alexa Fluor 546	ThermoFisher	A11010
Goat anti-rabbit Alexa Fluor 647	ThermoFisher	A21245
Goat anti-chicken Alexa Fluor 647	ThermoFisher	A21449
Goat anti-guinea pig Alexa Fluor 647	ThermoFisher	A21450
HaloTag ligand JF646	Promega	GA1120
**Bacterial and Virus Strains**		
DH10β	ThermoFisher	18297010
Stellar cells	Takara	636767
**Chemicals, Peptides, and Recombinant Proteins**		
Bovine Serum Albumin Fraction V	Roche	10735086001
Normal Goat Serum	Jackson Immunoresearch	005000121
Bovine Serum Albumin	Sigma	A3803
DAPI	Roche	10236276001
DMEM	Gibco	11995065
Fetal Bovine Serum	Gibco	16000044
Hanks’ Balanced Salt Solution	Gibco	14175095
HEPES	Sigma	H3375
Matrigel Membrane Matrix	ThermoFisher	CB-40234
Poly-D-lysine	Gibco	A38904-01
MEM Non-essential Amino Acid Solution	Sigma	M7145
Papain	Worthington Biochemical Corporation	LS003126
Opti-MEM	Gibco	31985070
Paraformaldehyde	Electron Microscopy Science	15714
Penicillin/Streptomycin	Corning	MT30002Cl
B-27 supplement	Gibco	17504044
Neurobasal A	Gibco	10888022
TransIT-2020	Mirus	MIR5400
Cytosine β-D-arabinofuranoside	Sigma	C6645
Versene	Gibco	15040066
FuGENE6	Promega	E2691
Benzonase nuclease	Sigma	E1014
Superscript IV Reverse Transcriptase	Invitrogen	18091050
In-Fusion Assembly system	Takara	638948
PowerUp SYBR Green Master Mix	Applied Biosystems	A25742
Prolong Gold Antifade Reagent	Invitrogen	P36930
**Experimental Models: Cell Lines**		
HEK293T	ATCC	CRL-11268
**Experimental Models: Organisms/Strains**		
C57/BL6J	Jackson Laboratories	000664
**Oligonucleotides**		
Various cloning primers	IDT	N/A

		
**Software**		
SnapGene	GSL Biotech	
NIS-Elements AR	Nikon	
ImageJ	National Institutes of Health	
Adobe Photoshop	Adobe	
Adobe Illustrator	Adobe	
Graphpad Prism 8.0, 9.0	Graphpad	
		
**Recombinant DNA**		
L202 rSyn mClover3-Homer1c	This study	
L202 rSyn mTagBFP2	This study	
L202 rSyn HaloTag-Syb2	This study	
L202 rSyn tdTomato-Gephyrin	This study	
pAAV nEF spCas9	This study	
pAAV SEP-GluA2 TKIT	Fang *et al*., 2021	
pAAV HA-Homer1c TKIT	This study	
pAAV HA-Bassoon TKIT	This study	
pAAV GFP-Gephyrin TKIT	This study	
pAAV tdTomato-Gephyrin TKIT	This study	

**Table 1.** CRISPR KI sequences, AAV and Lenti titering primers, and primers for estimating the tagging efficiency of the TKIT method.

Purpose	Sequence 5’-3’
Homer1c KI sgRNA1	TATTCAAGTGCACGTCGCGT
Homer1c KI sgRNA2	TTATTGTAGAGCGACACCAG
Gephyrin KI sgRNA1	TGCAACTCGTGGAGAGTGAG
Gephyrin KI sgRNA2	GAGAAACCTCCAGCAAGTCG
Bassoon KI sgRNA1	TGCGTGGACACGAGTCTTCG
Bassoon KI sgRNA2	TGGTCAAGGTGGGCAACCCT
AAV genomic titering forward	GGAACCCCTAGTGATGGAGTT
AAV genomic titering reverse	CGGCCTCAGTGAGCGA
Lenti genomic titering forward	CCACTGCTGTGCCTTGGAATGC
Lenti genomic titering reverse	AATTTCTCTGTCCCACTCCATCCAG
Homer1 WT TKIT F for tagging efficiency	GAGTTGCCTCCGGAAAAGATCTCGG
Homer1 WT TKIT R for tagging efficiency	CATGAGCTCGAGTGCTGAAGATAGG
Homer1 HA TKIT F for tagging efficiency	CCATACGATGTTCCAGATTACGCTG
Gphn WT TKIT F for tagging efficiency	TCCTGGCTCCTGTCAGTGCGGTG
Gphn WT TKIT R for tagging efficiency	GCAAGATTCCTGAAGCAGCTATCAC
Gphn GFP TKIT F for tagging efficiency	CTCTCGGCATGGACGAGCTGTAC
BSN WT TKIT F for tagging efficiency	CAACGAGGCCAGCCTGGAGG
BSN WT TKIT R for tagging efficiency	GGCCAGGAGCAGTAGCAGATGC
BSN HA TKIT F for tagging efficiency	CCATACGATGTTCCAGATTACGCTG
GAPDH	PrimeTime, IDT #MM.PT.39a.1
